# Coordinated Positioning Method for Shortwave Anti-Multipath Based on Bayesian Estimation

**DOI:** 10.3390/s22197379

**Published:** 2022-09-28

**Authors:** Tao Tang, Linqiang Jiang, Paihang Zhao, Na-e Zheng

**Affiliations:** Institute of Information Engineering, PLA Strategic Support Force Information Engineering University, Zhengzhou 450001, China

**Keywords:** shortwave, coordinated positioning, anti-multipath, prior information, deep learning

## Abstract

Coordinated positioning based on direction of arrival (DOA)–time difference of arrival (TDOA) is a research area of great interest in beyond-visual-range target positioning with shortwave. The DOA estimation accuracy greatly affects the accuracy of coordinated positioning. With existing positioning methods, the elevation angle’s estimation accuracy in multipath propagation decreases sharply. Accordingly, the positioning accuracy also decreases. In this paper, the elevation angle is modeled as a random variable, with its probability distribution reflecting the characteristics of multipath propagation. A new coordinated positioning method based on DOA–TDOA and Bayesian estimation with shortwave anti-multipath is proposed. First, a convolutional neural network is used to learn the three-dimensional spatial spectrogram to make an intelligent decision on the number of single and multiple paths, and to obtain a probability distribution of the elevation angle under multiple paths. Second, the elevation angle’s estimated value is modified using the elevation angle’s probability distribution. The modified elevation angle’s estimated value is substituted into a DOA pseudo-linear observation equation, and the target position’s estimated value is obtained using the matrix QR decomposition iteration algorithm. Finally, a TDOA pseudo-linear observation equation is established using the target estimate obtained in the DOA stage, and the coordinated positioning result is obtained using the matrix QR decomposition iteration algorithm again. Simulation results demonstrated that the proposed method had a stronger anti-multipath capability than traditional methods, and it improved the coordinated positioning accuracy of the DOA and TDOA. Measured data were used to validate the proposed method.

## 1. Introduction

Shortwave communication is an important means of long-distance communication and it has the characteristics of high destructibility and strong mobility. Shortwave signal source positioning has wide applications in both the military and civilian fields [1,2]. Target positioning based on position parameters is the main method for shortwave positioning. The position parameters mainly include direction of arrival (DOA), time of arrival, time difference of arrival (TDOA), and frequency difference of arrival. Usually, a single positioning parameter can be used to estimate the target position; however, a combination of multiple parameters can effectively improve the positioning accuracy [3]. DOA positioning and TDOA positioning are the two most representative shortwave positioning approaches. A combination of DOA and TDOA can significantly improve the accuracy of shortwave positioning.

For propagation within the visual range, researchers have proposed many algorithms for DOA positioning [4,5,6], TDOA positioning [7,8,9], and DOA–TDOA coordinated positioning [10,11,12]. However, these algorithms cannot be used directly in beyond-visual-range (BVR) shortwave communication. At present, there are few positioning algorithms for BVR shortwave targets. In [13], the authors proposed a TDOA positioning method for BVR shortwave targets based on a grid search. Their method has high positioning accuracy; however, it is a complex computational process. In [14], the authors proposed a TDOA positioning algorithm with gradient projection based on an ionospheric quasi-parabolic (QP) model. However, using the QP model requires many ionospheric parameters, which are difficult to obtain in practice. Moreover, the QP model has the limitation of only considering specific ionospheric reflections. Compared with the QP model, the ionospheric virtual height reflection model [15,16] only requires the ionospheric reflection virtual height as an input parameter, and different reflection virtual heights correspond to different ionospheric reflections. In [17], the authors proposed a method to conduct pseudo-linearization on the DOA and TDOA observation equations based on an ionospheric virtual height reflection model. They obtained solutions using an iterative matrix QR decomposition algorithm with simple calculations. However, this method only considers a single transmission path without considering the shortwave multipath effect in the practical process. In [18], the authors proposed a DOA–TDOA coordinated positioning method based on an ionospheric virtual height reflection model using two propagation paths without the need for a known ionospheric virtual height. However, this method requires a large amount of computation and requires the accurate arrival elevation angles of two propagation paths, which are difficult to achieve in practice.

The multipath effect under the ionospheric influence leads to difficulties in shortwave positioning, which has a great impact on the estimation of positioning parameters such as the arrival angle, and thus, the positioning accuracy. In actual shortwave positioning, the measurement error of the azimuth angle can reach about 3°, while the measurement error of the elevation angle can reach about 5–10°. Therefore, the elevation angle is not usually used for positioning. At present, data fusion is used to handle multipath data after the signal is received in the main methods designed for multipath scenarios [16,19,20]. However, these cannot be applied directly to the DOA or TDOA positioning scenarios in this study.

To summarize, in this paper, shortwave anti-multipath coordinated positioning with DOA–TDOA is addressed. To accurately reflect the characteristics of ionospheric reflection, the arrival elevation angle was simulated as a random variable with a Gaussian mixture model (GMM). First, a convolutional neural network (CNN) was used to learn the three-dimensional spatial spectrogram to identify the modes of single-path propagation and multipath propagation. The corresponding probability distribution of the elevation angle was obtained and used to modify the estimated value of the elevation angle. Then, a two-dimensional DOA pseudo-linear observation equation was established based on the azimuth estimate and the elevation angle’s modified estimate. The matrix QR decomposition iteration algorithm proposed in [17] was used to obtain the DOA positioning results. Finally, a TDOA pseudo-linear observation equation was established based on the DOA positioning results, and the final positioning results were obtained using the matrix QR decomposition iteration algorithm again.

The remainder of this paper is as follows: Section 2 introduces the impact caused by the multipath problem and the observation model. Section 3 proposes the improved method. Section 4 shows the results of the simulation experiments. Section 5 shows the results of the measured data, and Section 6 summarizes the paper.

## 2. Problem Statement and Observation Models

### 2.1. Multipath Problem

**Figure 1:**
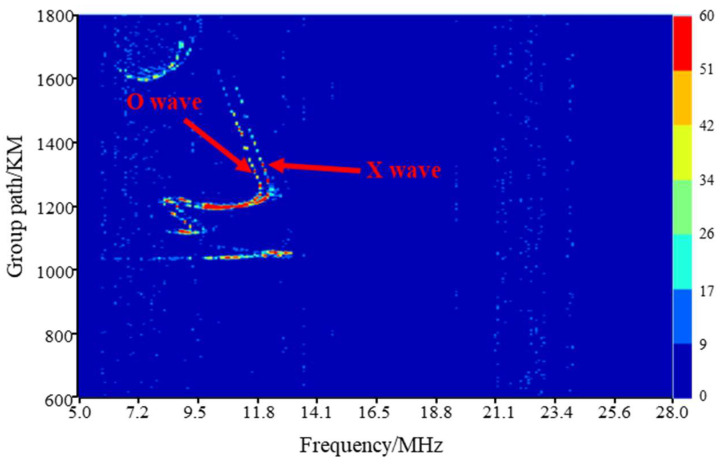
An example of an ionization map in ionospheric passive oblique detection.

The ionospheric signal channel varies randomly with space, time, and frequency, which has an impact on transmitted signals such as multipath fading and polarization fading. The electromagnetic wave incident in the ionosphere is divided into an ordinary (O) mode and unusual (X) mode. These two modes correspond to different paths and generate multipath fading. Ionospheric passive oblique detection technology can be used to obtain the ionospheric information regarding the reflection points between the transmitting and receiving stations. The high frequency radio signal is transmitted by the transmitting station, and the delay in the propagation time is calculated by the signal received by the receiving station, so as to infer the characteristics of the ionosphere. Figure 1 shows the result of passive oblique detection. The abscissa in Figure 1 represents frequency and the ordinate represents the group path. The colors indicate the electron concentrations. It can be observed that when the frequency is between 10 and 12 MHz, there are two propagation modes that correspond to the O and X waves. Therefore, when the communication frequency falls within this frequency range, there are multiple propagation paths that affect signal reception.

**Figure 2:**
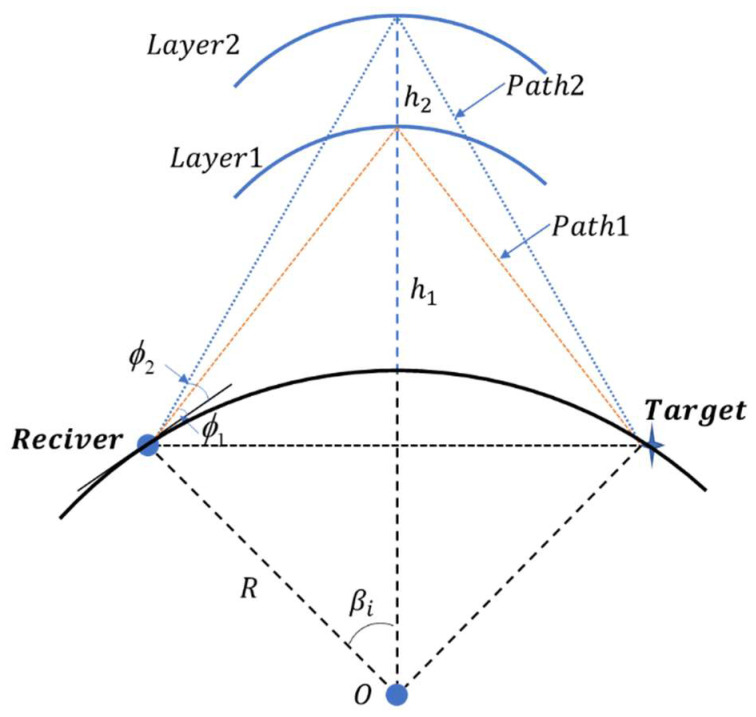
Geometric illustration of multipath propagation.

Figure 2 shows a geometric illustration of multipath propagation. The electromagnetic wave reaches the receiving station after it is transmitted through the ionosphere. Because of the layered structure of the ionosphere, each layer corresponds to a different reflection height. The electromagnetic wave can reach the receiving station through different reflection heights that correspond to different paths. The elevation angles of the signals that reach the receiving station through different paths are different [15]. Target positioning under two paths was investigated in this study.

**Figure 3:**
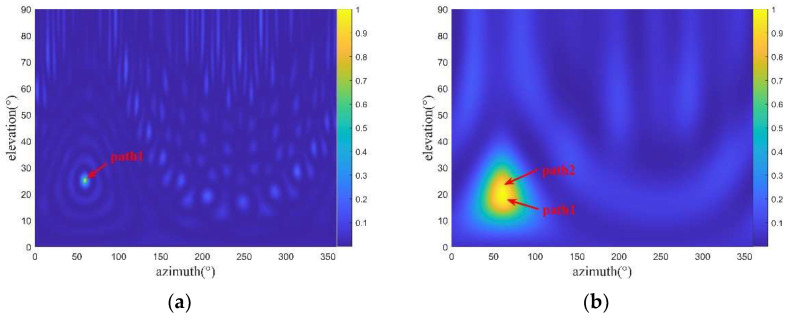
Spatial spectrogram examples of a single path (**a**) and two paths (**b**) (top view).

Multipath propagation in the spatial spectrogram is manifested by multiple spectral peaks at the same azimuth. Therefore, angle estimation ambiguity may occur [16]. Figure 3 shows spatial spectrogram examples of a single path and two paths. In the case of two paths, the spectral peaks are formed at two elevation angles, and the two spectral peaks are not easy to separate. Therefore, the angle estimation accuracy is affected.

**Remark** **1.** *Only one single path and two paths are taken as examples to illustrate the influence of multipath on the spatial spectrogram. When the number of paths increases, the spectral peak of spatial spectrum will increase correspondingly; however, the case for a larger number of paths is very rare [20]*.

When the fading amplitude follows the Rayleigh distribution, the probability distribution of the multipath time delay follows a normal distribution [21]. Inspired by this phenomenon, in this study, the authors modeled the single-path arrival elevation angle as a Gaussian distribution, with the true value as the mean. Figure 2 shows that the reflection height corresponds to the elevation angle. Because the ionosphere is time varying, the reflection height also varies, which results in a change in the elevation angle. Therefore, a Gaussian distribution model of the elevation angle can reflect the ionospheric change. When the elevation angles under the two paths are simulated as a GMM, their mean values are the true values at the corresponding reflection heights. The multipath elevation angles are modeled as a GMM to determine target positioning with multipath.

### 2.2. Array Receiving Signal Model

For an assumed circular array with *p* elements, there are *q* signals that have incident angles $\Theta_{1},\Theta_{2},\ldots ,\Theta_{q}$ in the array, where $\Theta_{i}=(\theta_{i},\phi_{i})$, and $\theta_{i}$ Lining $\phi_{i}$ are the arrival angle and elevation angle of the *i*th signal, respectively. The arrival azimuth refers to the angle between the incident direction of the signal and the north direction of the local observation station. The elevation angle refers to the angle between the incident direction of the signal and the surface plane of the observation station. The output of the *k*th element in the antenna array can be expressed as

(1) $$x_{k}(t)=\sum_{i=1}^{q}g_{ki}s_{i}(t-\tau_{ki})+n_{k}(t)$$

where $s_{i}(t)$ denotes the *i*th incident signal in the array, $g_{ki}$ denotes the complex gain of the kth array element in the *i*th signal array, $n_{k}(t)$ denotes the additive noise in the kth array element, and $\tau_{ki}$ denotes the time delay of the signal reaching the array element relative to the reference point.

It is assumed that the array elements are isotropic and there are no impacts, such as channel inconsistency and mutual coupling. Therefore, $g_{ki}=1$, and the received signal model of the array is given by

(2) X=A(Θ)S+N

In Equation (2), $X={\left[x_{1}(t),\ldots ,x_{p}(t)\right]}^{T}\in C^{p\times 1}$ is the array output vector. $N={\left[n_{1}(t),\ldots ,n_{p}(t)\right]}^{T}\in C^{p\times 1}$ is the additive noise vector of the array. $S={\left[s_{1}(t),\ldots ,s_{q}(t)\right]}^{T}\in C^{q\times 1}$ is the signal source vector. $A(\Theta )=[a(\Theta_{1}),\ldots ,a(\Theta_{q})]\in C^{p\times q}$ is an array flow pattern matrix, where $a(\Theta_{i})={\left[e^{-j\omega_{o}\tau_{1i}},\ldots ,e^{-j\omega_{o}\tau_{1p}}\right]}^{T}\in C^{p\times 1}$ is the array direction vector, and $\omega_{o}$ is the carrier frequency of the signal.

**Remark** **2.** A*is the array manifold matrix, which is related to the shape of the array and the direction of the signal. In practice, the shape of the antenna array will not change once it is fixed, so*A*is closely related to the direction of the signal. In practice, the self-adjustment of the antenna array can ensure*A*can be used for angle estimation, and there will be no ill-conditioned matrix.*  N *stands for additive noise. The larger it is, the lower the accuracy of angle estimation. When it reaches a certain level, angle estimation cannot be carried out. In addition, *N *does not have a substantial impact on the structure of matrix* A, *and* N *only has an impact on the estimation accuracy.*

Under the narrowband model defined in Equation (2), the DOA estimation based on the multiple signal classification (MUSIC) algorithm is given by

(3) $$\Theta_{MUSIC}=\underset{\Theta }{\arg\max}(1/(a^{H}(\Theta )U_{N}U_{N}^{H}a(\Theta )))$$

In Equation (3), $U_{N}\in C^{p\times (p-q)}$ is the noise subspace in the MUSIC algorithm.

### 2.3. MUSIC Algorithm

The MUSIC algorithm is a classical direction of arrival estimation algorithm, and the main steps are as follows:

(1) Calculate the covariance matrix of array output data based on Equation (2);
(2) Eigenvalue decomposition is performed on the covariance matrix obtained in step (1) to obtain the signal subspace $U_{S}$ and noise subspace $U_{N}$;
(3) Search the angle corresponding to the maximum value of Equation (3), which is the angle estimate value.

### 2.4. Positioning Solution Algorithm

The positioning scenario in this study is the same as that in [16], with *N* DOA positioning observation stations and *M* TDOA positioning observation stations, which are denoted as $u_{1},u_{2},...,u_{N},u_{N+1},...,u_{N+M}$.

The positioning algorithm is divided into two stages. In the first stage, the pseudo-linear observation equations of the azimuth and the elevation angle are established, as shown in Equation (4). According to the geographical constraints of the earth surface and the algebraic relationship between the auxiliary variables $||u|{|}_{2}^{2}$ and the target position $u\in R^{3\times 1}$, the equality constraint shown in Equation (5) can be obtained. u is the target position vector in the earth-centered earth-fixed coordinate system. The estimation criterion with a double quadratic equality can be obtained by combining Equations (4) and (5). Then, the DOA stage estimate can be obtained using the matrix QR decomposition iteration algorithm [16] as follows:

(4) $$\begin{array}{l} B_{\theta }u=b_{\theta }\\ B_{\phi }\left[\begin{array}{c} u\\ ||u|{|}_{2}^{2} \end{array}\right]=b_{\phi } \end{array}$$

(5) $$\left\{\begin{array}{c} t_{u}^{T}A_{1}t_{u}^{T}=R_{e}^{2}\\ t_{u}^{T}A_{2}t_{u}^{T}+c_{1}^{T}t_{u}^{}=0 \end{array}\right.$$

where $B_{\theta }\in R^{N\times 3}$, $B_{\phi }\in R^{N\times 4}$, $t_{u}^{}\in R^{4\times 1}$ and $R_{e}$ is the equatorial radius of the earth and e is the first eccentricity of the earth. In Equation (5)

(6) $$\begin{array}{l} B_{\theta }[i,:]={\left[s_{i,1}\cos(\theta_{i})-s_{i,2}\sin(\theta_{i})\right]}^{T},b_{\theta }[i,:]={\left(s_{i,1}\cos(\theta_{i})-s_{i,2}\sin(\theta_{i})\right)}^{T}u_{i}\\ B_{\phi }[i,:]={\left[2u_{i}^{T},-1\right]}^{T},b_{\phi }=||u_{i}|{|}_{2}^{2}-||u-u_{i}|{|}_{2}^{2},i=1,2,...,N\\ A_{1}=diag\{1,1,1/\left(1-e^{2}\right),0\},A_{2}=diag\{1,1,1,0\}\\ c_{1}={\left[0,0,0,-1\right]}^{T}\\ t_{u}^{}=\left[\begin{array}{c} u\\ ||u|{|}_{2}^{2} \end{array}\right] \end{array}$$

$s_{i,2}={\left[-\cos(\omega_{i,1})\sin(\omega_{i,2}),-\sin(\omega_{i,1})\cos(\omega_{i,2}),\cos(\omega_{i,2})\right]}^{T}$, $s_{i,1}={\left[-\sin(\omega_{i,1}),\cos(\omega_{i,2}),0\right]}^{T}$, $\omega_{i,1},\omega_{i,2}$ are the longitude and latitude of the *i*th observation station, respectively.

In the second stage, the pseudo-linear equation of the time difference is established based on the DOA estimate, as shown in Equation (7). Similarly, Equations (5) and (7) are combined to construct an estimation criterion that contains the double quadratic equality. The matrix QR decomposition iteration algorithm is used to obtain the final positioning results [16].

(7) $$\begin{array}{l} B_{\tau }\left[\begin{array}{c} u\\ ||u|{|}_{2}^{2} \end{array}\right]=b_{\tau }\\ B_{\tau }[m-1.:]=\left[2a_{m}^{2}u_{N+m}^{T},-a_{m}^{2}\right],b_{\tau }[m-1,:]=4R_{0}^{2}\mu_{m}^{2}+a_{m}^{2}(||u_{N+m}^{}|{|}_{2}^{2}-4R_{0}^{2})\\ m=2,3,...,M \end{array}$$

where $B_{\tau }\in R^{(M-1)\times 4}$, $a_{m}^{}=2R_{0}(R_{0}+h_{m})$, $\mu_{m}^{}$ is defined in Equation (73) of [16]. $R_{0}$ is the average radius of the earth.

## 3. Improved Method

### 3.1. Path Number Discrimination and the Elevation Angle’s Prior Distribution Learning Based on the CNN

**Figure 4:**
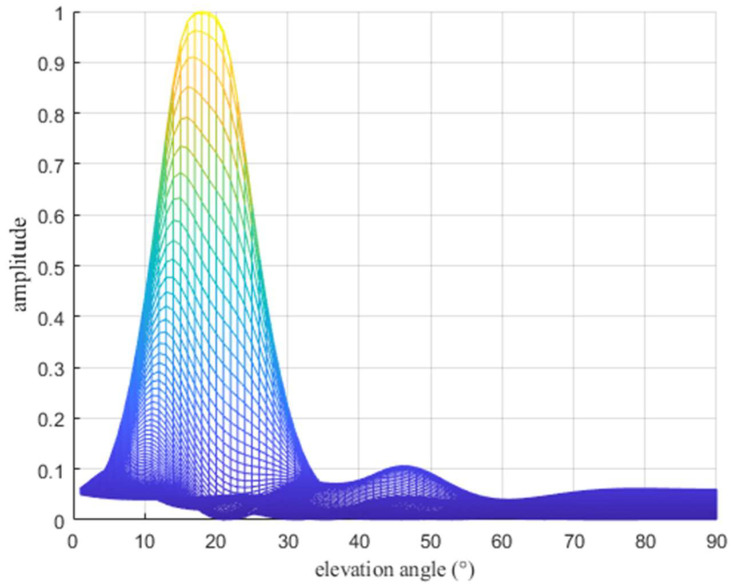
Illustration of spatial spectrograms with elevation angles of 16.4° and 20.5° under two paths (elevation view).

The CNN is a type of feedforward neural network that contains convolutional computation and a deep structure [22,23]. It is a representative deep learning network and widely used in the field of image processing. Its learning technology has matured gradually. In this study, when the two paths are close to each other, the two spectral peaks are merged into one spectral peak in the spatial spectrogram, as shown in Figure 4. Traditional spectral peak search techniques cannot distinguish a single path from two paths; hence, only one spectral peak can be obtained rather than the elevation angles of two paths.

In this study, the CNN was used to detect and identify spatial spectrograms to discriminate between a single path and multiple paths. In the multipath case, the CNN was used to obtain the elevation angle of each path.

#### 3.1.1. Path Number Discrimination

The problem of path number discrimination based on spatial spectrograms belongs to an image classification problem with supervised learning. In this study, 10,000 spatial spectrogram images with different numbers of paths were generated with an elevation angle range of 5° to 70°. The number ratio between the single path and the two paths was approximately 1:1. The spatial spectrogram contained RGB images of 256 × 256 pixels.

**Remark** **3.** *When there are multiple signals, there are many possible combinations of signals and the number of paths, which will lead to the very complex training process of neural network and a large amount of training data is required. In addition, based on the theory of array signal processing [24], when the arrival direction of multiple signals is different, multiple signals can be reduced into a single signal by dimensionality reduction. Therefore, each signal can be processed in this way. By using this method, the accuracy will not decrease, but the computation can be greatly reduced.*

**Remark** **4.** *The network used in this paper can be used to identify the number of paths under more paths, and only needs to increase the corresponding training data. However, the case for a larger number of paths is very rare [21] so this paper focuses on two common cases: single path and two paths.*

**Figure 5:**
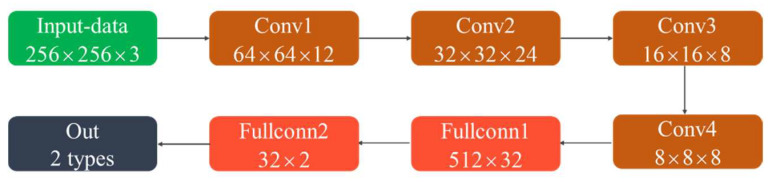
Structure of the CNN for path number discrimination.

The CNN structure is shown in Figure 5. It contained four convolutional layers and two fully connected layers. The convolutional layer is used to extract spatial spectral features. The fully connected layer plays the role of mapping the learned feature representation to the label space of samples. The activation function was the ReLU function. The loss function was the BCELoss function. To prevent overfitting, an early stop mechanism was used in the training process. If the loss value of the validation set did not decrease for 10 consecutive times, the training was stopped.

**Figure 6:**
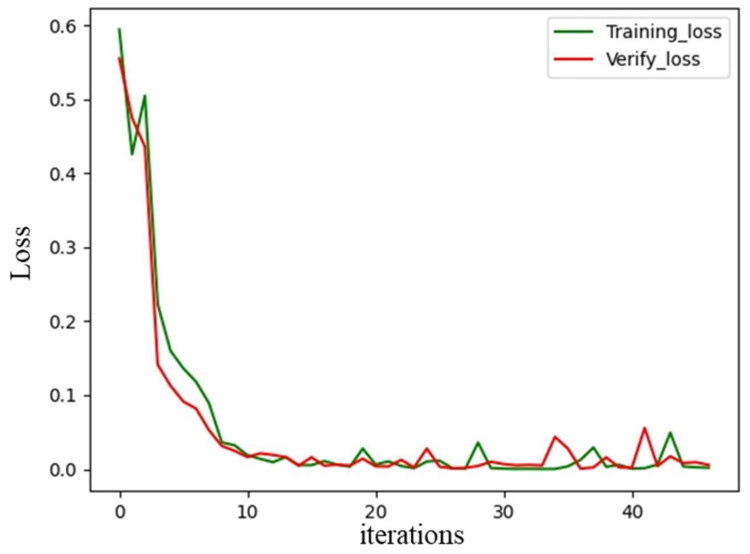
Loss curve of the path number discrimination model.

The loss curves of the training set and validation set are shown in Figure 6. The loss values of the training and validation sets maintained the same decreasing trend, and there was no overfitting during the training process. A total of 2000 spatial spectrograms were used as the test set. The accuracy of path number discrimination was 99.8%. Therefore, we can use the trained network to determine the number of paths.

#### 3.1.2. Search for Multipath Spectral Peaks

**Figure 7:**
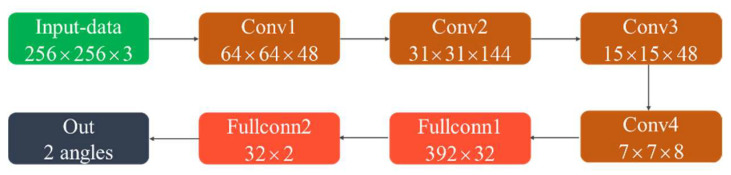
CNN structure used in the elevation angle calculation.

**Figure 8:**
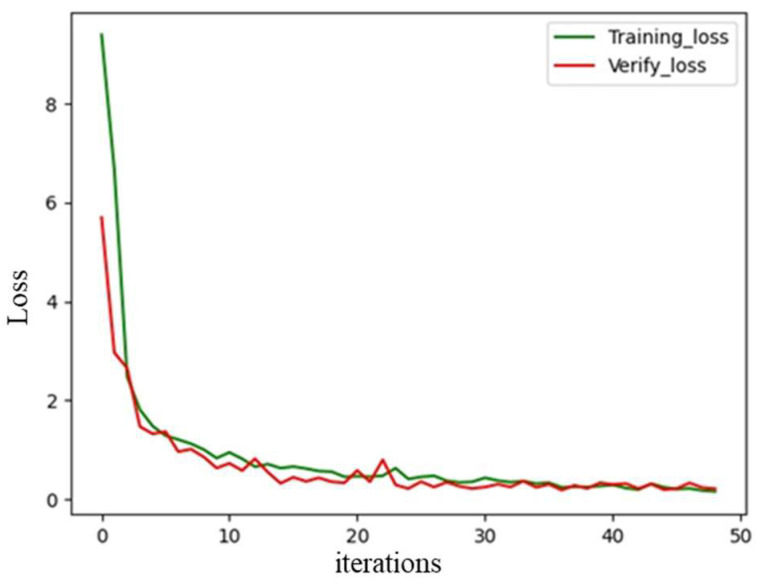
Loss curve of the elevation angle calculation model.

After the number of paths was determined, the search for the spectral peak in the spatial spectrogram was performed using a regression method based on the CNN. The regression network model structure is shown in Figure 7. The convolutional layer dimension was added in the model structure based on the path number discrimination model to improve the characteristic extraction capability of the model. The model output was modified from the classification result to the regression result of the elevation angle. The loss function was the MSELoss function. A total of 10,000 spatial spectral spectrogram images of the two paths were generated as the training data with an elevation angle range of 5° to 70°. The early stop mechanism was the same as the network shown in Figure 5. The loss curve in the training process is shown in Figure 8. The decreasing trend of the loss value in the training set was consistent with that in the validation set. There was no overfitting in the training process. A total of 1000 randomly generated spatial spectrogram images were used as the test set. When the angle error was within 1.5°, the prediction accuracy was acceptable. The prediction accuracy rate of the elevation angle is defined as the ratio of the number of correctly predicted spatial spectral spectrograms to the number of total spatial spectral spectrograms. It was 94.75%, which demonstrated satisfactory model performance that met the expected requirements.

#### 3.1.3. Elevation Angle’s Prior Distribution Learning

**Figure 9:**
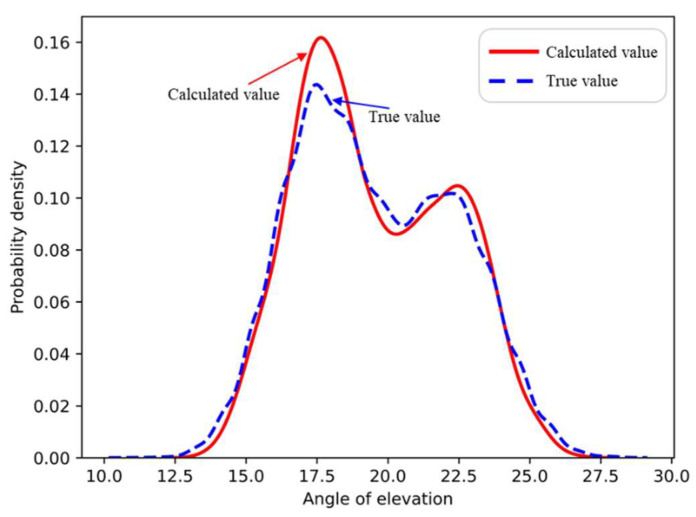
Comparison of elevation angle distribution learning.

A simulation was conducted to generate 10,000 spatial spectrogram images that included a single path and two paths for a specific region. The elevation angles of the two paths followed a GMM. First, the number of paths was determined by the path discrimination model to obtain the numbers of single-path and two-path spectrograms, which were 3823 and 6177, respectively. For the single-path spatial spectrograms, the elevation angle was obtained using a traditional search method for the spectral peak. For the two-path spatial spectrums, the network shown in Figure 7 was used to determine the elevation angle, and the probability distribution of the elevation angle was also collected. Figure 9 shows the result of the comparison between the actual probability distribution of the elevation angle and the probability distribution obtained by CNN learning. As shown in Figure 9, neural network learning helped us to obtain an important characteristic, that is, the elevation angles of the two paths followed a GMM. Therefore, the network met the expected requirements.

### 3.2. Estimate Modification of the Elevation Angle Based on Prior Information

Under the influence of multipath fading, the elevation angle ϕ of the same target is no longer a fixed value but a random variable that varies with the direction measurement’s time and site, denoted by ϕ(r,t), where *r* is the location of the measurement station and *t* is time. Different from traditional methods in which the elevation angle is considered as a deterministic parameter for direction measurement, in this paper, Bayesian estimation theory was introduced and the elevation angle distribution under the ionospheric multilayer reflection was considered as prior information to estimate the maximum posterior probability of the elevation angle as follows:

(8) $$P(\phi |X)=\frac{P(X|\phi )P(\phi )}{P(X)}$$

where X is the array observation vector defined by Equation (2), P(ϕ|X) is the posterior probability, P(X|ϕ) is the likelihood function of the elevation angle with respect to the observation vector, and P(ϕ) is the prior probability distribution of the elevation angle. Equation (8) can be simplified as follows:

(9) P(ϕ|X)=ηP(X|ϕ)P(ϕ)

where

(10) $$\eta ={\left(P(X)\right)}^{-1}=\frac{1}{\sum_{A}P(X|\phi )P(\phi )}$$

The prior distribution information of the elevation angle can be used to modify the posterior probability to obtain the modified posterior probability. This idea is important and provided by Equation (9). According to this idea, the prior distribution of the elevation angle can be used to modify the elevation angle’s estimated value obtained using Equation (3).

(11) $$\phi_{MUSIC}=\underset{\phi }{\arg\min}(a^{H}(\Theta )U_{N}U_{N}^{H}a(\Theta ))\;P(\phi )$$

It should be noted that the elevation angle’s correction only involved multiplying the angle’s estimate by a coefficient, so that the increase in the computational effort was limited. Although the prior probability distribution of the elevation angle requires a large amount of data analysis, the computation is an offline process. Therefore, only one deep learning calculation is needed to obtain the distributions for actual positioning.

As shown in Figure 2, the calculation method of the azimuth and the elevation angle of the signal that passes each path to arrive at the *i*th DOA observation station is as follows:

(12) $$\theta_{i}=\arctan\left(\frac{u_{x}-u_{ix}}{u_{y}-u_{iy}}\right),\frac{R}{\sin\left(\frac{\pi }{2}-\phi_{i}-\beta_{i}\right)}=\frac{R+h_{i}}{\sin\left(\frac{\pi }{2}+\phi_{i}\right)},i=1,2,...,N$$

where *R* is the average radius of the earth, and u were $u_{i}$ are the coordinates of the target and *i*th observation station, respectively, in the earth-centered earth-fixed coordinate system.

The modified estimates of the azimuth and the elevation angle were obtained using Equation (3) and (11). The estimates were substituted into Equation (4) to obtain the pseudo-linear equations of the azimuth and the elevation angle. The matrix QR decomposition iteration algorithm was used to obtain the DOA stage positioning results.

According to Figure 2, the propagation distance of each path can be expressed as follows:

(13) $$d_{m}=2\sqrt{{\left(R\sin(\beta_{m})\right)}^{2}+{\left(R-R\cos(\beta_{m})+h_{m}\right)}^{2}}\;m=1,2,\ldots ,M$$

Therefore, the difference between the arrival time of the signal at the *m*th observation station and the arrival time at the first observation station can be calculated as

(14) $$\tau_{m}=\frac{2}{c}(d_{m}-d_{1})$$

Considering $\beta_{m}=\arcsin\left(\left||u-u_{m}||/(2R\right)\right)$ and that this calculation is related to the target position, Equation (14) cannot be pseudo-linearized directly. In this case, the target estimate of the DOA in the first stage can be considered as a known value in the TDOA stage to obtain the pseudo-linear equation shown in Equation (7) and obtain the final results using the matrix QR decomposition iteration algorithm.

## 4. Simulation Studies

Simulations were conducted to verify the positioning performance of the proposed method. The default settings of the simulation parameters included the following. The longitude and latitude of the radiation source were 134° and 34°, respectively. There were seven shortwave observation stations, among which the first three had the DOA positioning system and the last four had the TDOA positioning system. Their longitudes, latitudes, and corresponding ionospheric reflection heights are shown in Table 1. The observation errors of the azimuth, elevation angle, TDOA, and ionospheric reflection height all followed Gaussian distributions with zero means and were independent of each other. Their covariance matrices were

(15) $$\begin{array}{l} \Omega_{\Theta }=blkdiag\{\Omega_{\theta },\Omega_{\phi }\}=\sigma_{\theta }^{2}I_{2N}\\ \Omega_{\tau }=\sigma_{\tau }^{2}R_{M-1}\\ \Omega_{h}=\sigma_{h}^{2}I_{N+M} \end{array}$$

where the diagonal elements of the matrix $R_{M-1}$ were 1, and all other elements were 0.5. blkdiag{⋅} is a block-diagonal matrix formed from the matrices or vectors. The root mean square error of positioning was the measurement standard of positioning accuracy. The calculation formula for the root mean square error of positioning is given by

(16) $$RMSE=\sqrt{\sum_{i=1}^{K}\frac{||\hat{u}-u|{|}_{2}^{2}}{K}}$$

where *K* denotes the number of Monte Carlo simulation runs. *K* takes 5000 in the simulation.

**Figure 10:**
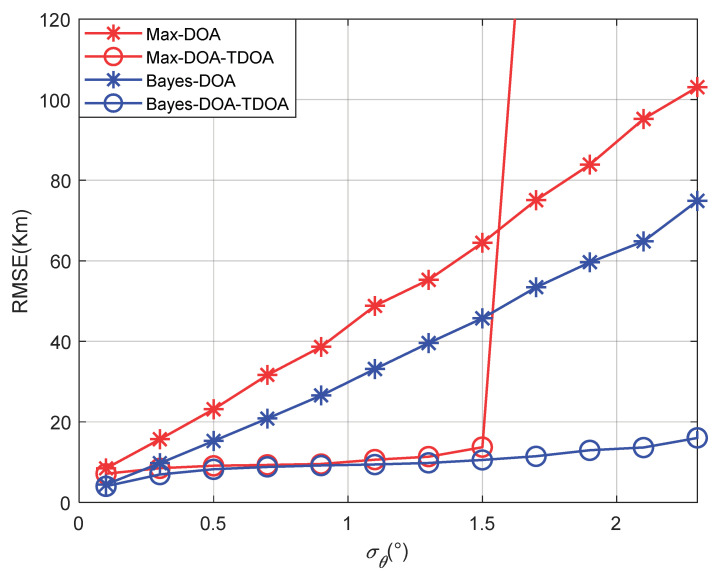
The positioning accuracy varies with the angle’s standard deviation.

**Figure 11:**
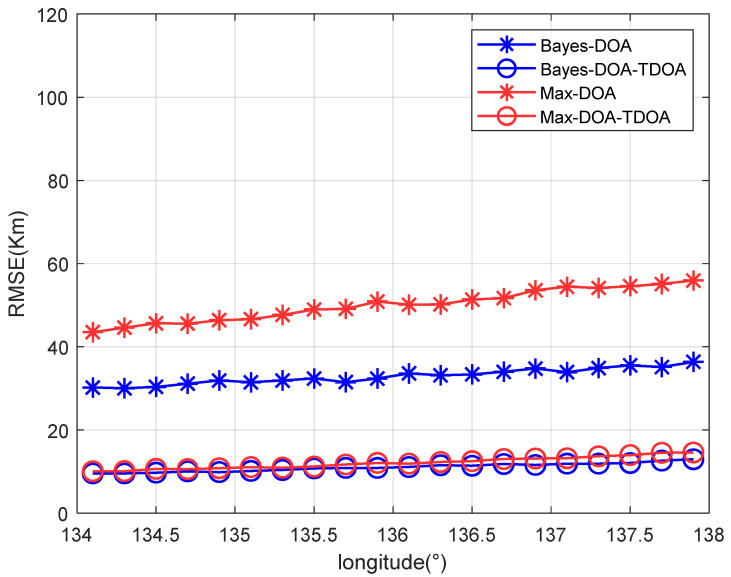
The positioning accuracy varies with the target’s accuracy.

Figure 10 shows that the results for the positioning accuracy vary with the standard deviation of the angle’s error with a single path. Other parameters were set as $\sigma_{\tau }^{}=(0.3/c)/s$ and $\sigma_{h}^{}=2\mathrm{km}$, where the elevation angle’s search step was 0.01°. “Max” means that the spectral peak with the largest spatial spectrum was selected as the angle’s estimate when the MUSIC algorithm was used. Figure 10 shows that the traditional positioning algorithm failed when the angle error was greater than 1.5°, which resulted in a sharply increasing positioning error. By contrast, the proposed method improved the estimation accuracy of the elevation angle; therefore, the failure threshold of the algorithm was raised. Figure 11 shows that the results for the positioning accuracy vary with the target’s longitude under a single path. As shown in Figure 11, when the distance between the observation station and the target increased, the positioning accuracy decreased, mainly because DOA positioning was sensitive to the distance between the target and the observation station. However, the accuracy did not decrease greatly. Therefore, the proposed method has a certain generalization capability at the location of the radiation source.

Table 1 shows the ionospheric reflection height; hence, the reflection elevation angle can be determined. The elevation angle of the second path was given by the elevation angle difference of 1°, 3°, 5°, 7°, and 10° in Table 1. In the case of two paths, two methods are usually used to estimate the angle, and these are called traditional methods. The first method selects the angle corresponding to the maximum peak of the two spectral peaks in the spatial spectrum as the angle’s estimated value, denoted by “Max.” The second method uses the average value of the angles corresponding to the two spectral peaks in the spatial spectrum as the angle’s estimated value, denoted by “Average.” The data given by the proposed method are denoted as “Bayes.”

**Figure 12:**
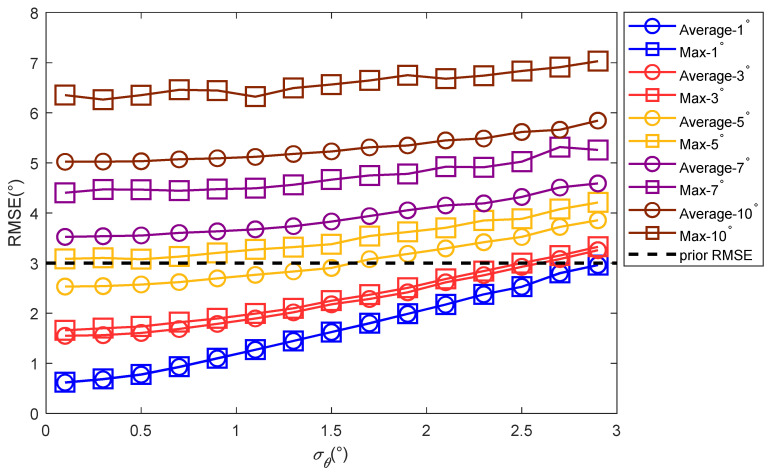
The elevation angle’s estimate accuracy varies with the angle’s standard deviation for the traditional methods.

**Figure 13:**
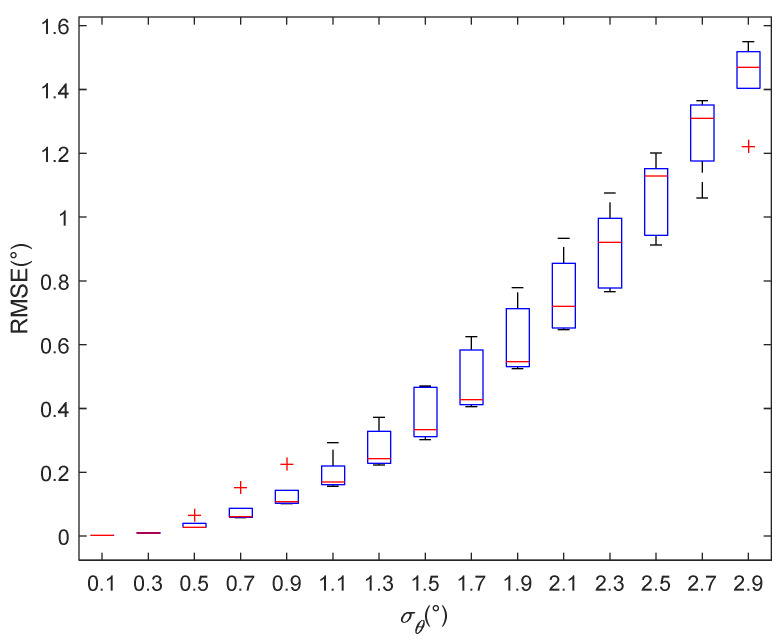
The elevation angle’s estimate accuracy varies with the standard deviation of the angle’s error for the proposed method.

Figure 12 and Figure 13 show the root mean square errors of the estimated values of the elevation angles given by the traditional methods and proposed method when the elevation angles were different. Figure 12 shows that when the difference between the elevation angles of the two paths was greater than 5°, the angle’s estimation accuracy was less than 3°, and the positioning algorithm failed. Figure 13 shows that the elevation angle’s estimation accuracies for the proposed method were all below 2° under various angle differences, which indicates that the proposed method had a certain anti-multipath effect.

**Figure 14:**
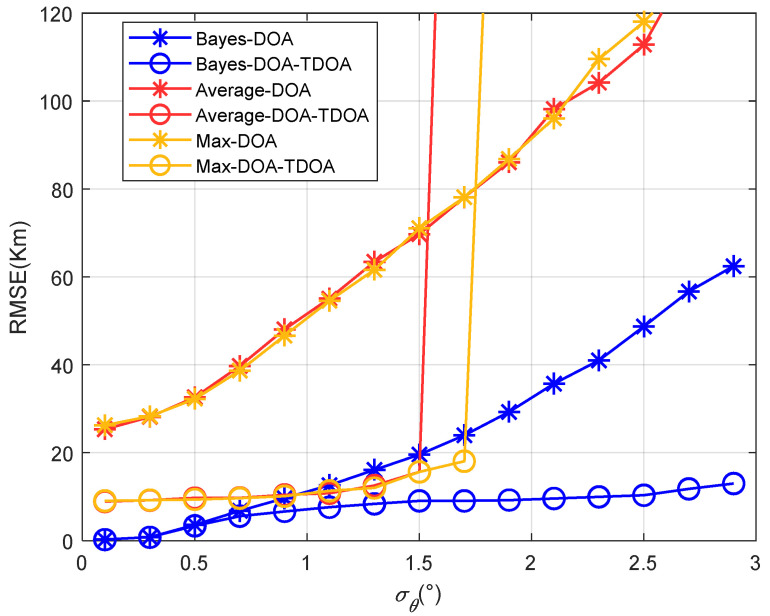
The positioning accuracy varied with the standard deviation of the angle’s error when the multipath elevation angle difference was 1°.

**Figure 15:**
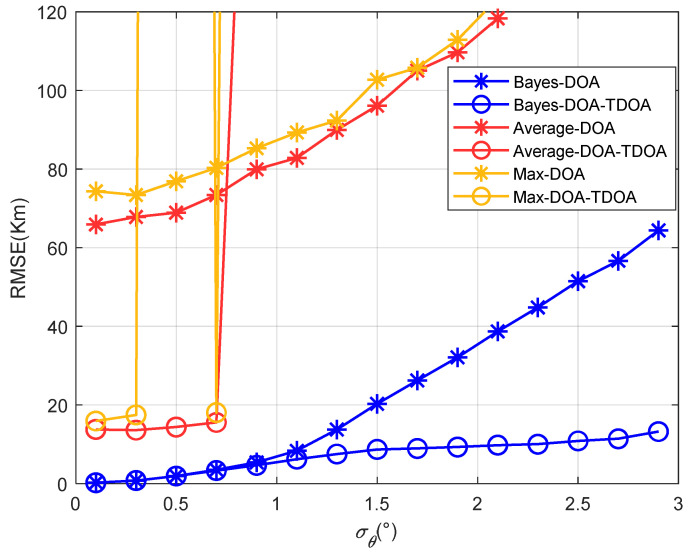
The positioning accuracy varied with the standard deviation of the angle’s error when the multipath elevation angle difference was 3°.

Figure 14 and Figure 15 show that the results for the positioning accuracy of the traditional methods and proposed method varied with the standard deviation of the angle’s error when the differences between the elevation angles of the two paths were 1° and 3°, respectively. As the angles’ difference between the two paths increased, the positioning error of the traditional methods gradually increased, and the threshold value was reduced. This shows that when the difference between the angles of the two paths was large, the traditional method could not position accurately. Moreover, when the DOA positioning error was large in the first stage, the accuracy of the proposed coordinated positioning was even lower than that of the standalone DOA positioning. This indicates that when the DOA accuracy was low, coordinated positioning was not meaningful. In this case, the accuracy of standalone positioning was better than that of coordinated positioning. It also indicates that the accuracy of coordinated positioning was not always better than that of standalone positioning.

**Figure 16:**
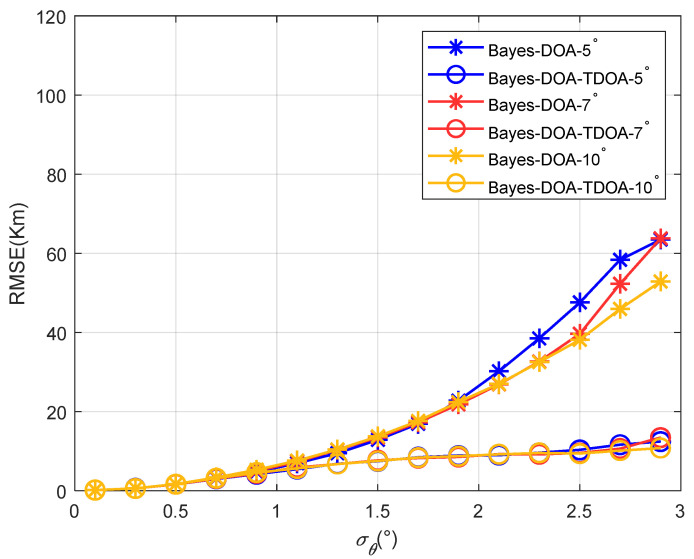
The positioning accuracy varied with the standard deviation of the angle’s error when the multipath elevation angles’ differences were 5°, 7°, and 10°.

Figure 16 shows the positioning accuracy varied with the standard deviation of the angle’s error when the elevation angles’ differences were 5°, 7°, and 10° for the proposed method. Because the traditional methods were not effective at this point, they are not shown in the figure.

Figure 14, Figure 15 and Figure 16 show that as the angle difference increased, the proposed method maintained high accuracy. Therefore, the proposed method had a strong anti-multipath function. This is because the prior information for the elevation angle is related to the position and time of the observation station, but is irrelevant to the path’s state. Therefore, prior information regarding the elevation angle can be used to correct the elevation angles under different angle differences.

## 5. Measured Data

The ionospheric multipath reflection model and its variation trends with respect to time were validated using measured data. The transmitting station used in the experiment was a radio station in Urumqi (87° E, 43° N), Xinjiang, China. The receiving station was a 20-channel shortwave direction measurement array located in Zhengzhou (113° E, 34° N), Henan Province, China. The experimental parameters are shown in Table 2.

**Figure 17:**
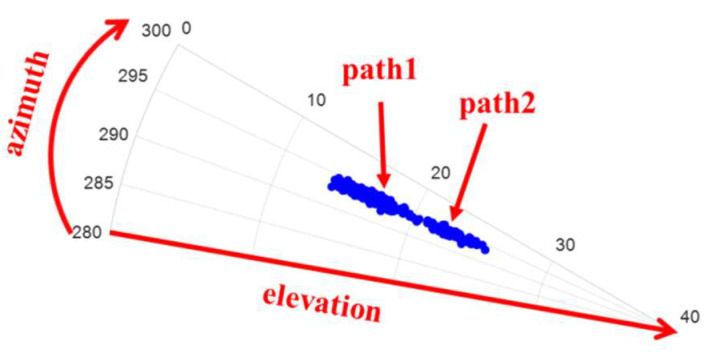
Local amplification of signal directions.

Figure 17 shows an illustration of the arrival angle’s estimate of the transmitting station obtained using the receiving array. In the signal direction, the elevation angle varied greatly, and the multipath effect existed. Based on the figure, the elevation angles of the two paths could not be separated accurately. Therefore, the estimation accuracy of the elevation angle was reduced because of the existence of the multipath effect.

**Figure 18:**
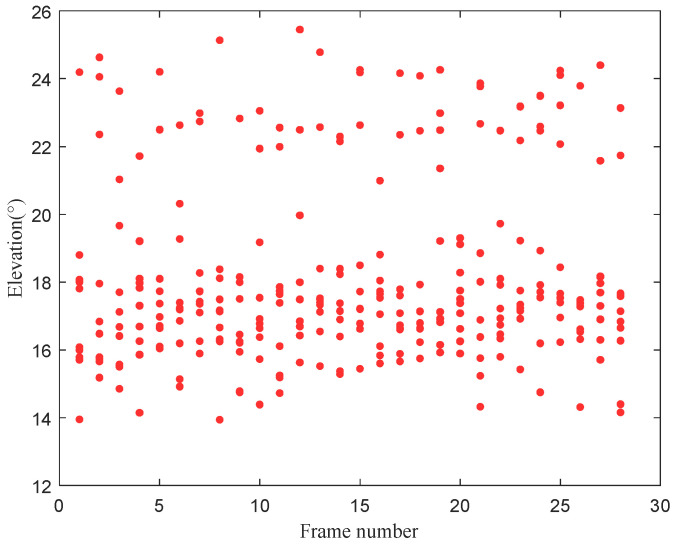
Illustration of the signal’s elevation angle varying with time.

**Figure 19:**
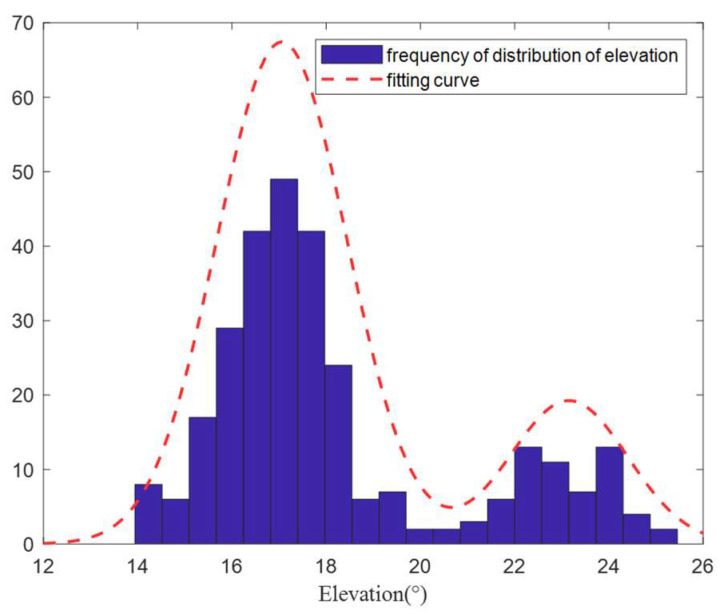
Statistical results for the elevation angle.

Figure 18 shows the time-varying results of the elevation angle of the signal. The difference between the elevation angles of the two paths was small at some moments, whereas the difference was large at other moments. Figure 19 shows the distribution of the signal’s elevation angle. The elevation angle distribution of the two paths was approximately a GMM, thus verifying the previous analysis and the validity of this study.

## 6. Conclusions

In this paper, DOA–TODA coordinated positioning for shortwave radiation sources was addressed based on prior information regarding the distribution of the elevation angles. First, a CNN was used to determine the number of single and multiple paths. The probability distribution of the elevation angle with the corresponding number of paths was learned. Then, the DOA and TDOA observation models were constructed according to the ionospheric virtual reflection model. The elevation angle’s estimated value was modified using the elevation angle’s prior information. An optimization model with double quadratic equality constraints was constructed according to the pseudo-linear equations of the azimuth, the elevation angle, and TDOA. The matrix QR decomposition iteration algorithm was used to solve the model.

Simulations were conducted for the single-path and two-path cases. The simulation results demonstrated that compared with traditional methods, the proposed method achieved better positioning accuracy when the angle error was large, and anti-noise performance improved with strong anti-multipath performance. Moreover, the proposed method generalized the target position. Finally, the measured data were used to validate the proposed method. It should be noted that in this paper, only DOA–TDOA coordinated positioning for stationary targets was addressed. A future study needs to be conducted for moving targets.

## Figures and Tables

**Table 1:** Longitude, latitude, and ionospheric reflection virtual height of the observation station.

Observation Station	Longitude (°)	Latitude (°)	Ionospheric Reflection Virtual Height (km)
DOA-1	116.23	40.22	340.00
DOA-2	112.54	33.00	390.00
DOA-3	116.00	29.71	370.00
TDOA-1	123.47	41.80	310.00
TDOA-2	114.54	38.04	350.00
TDOA-3	114.03	30.58	385.00
DOA-1	116.23	40.22	340.00

**Table 2:** List of experimental parameters.

Parameter	Signal Modulation Type	Distance	Azimuth	Radius-to-Wavelength Ratio
Value	AM	2447 km	294.1°	2.5

## Data Availability

Not applicable.
